# Factors that Contribute to Resident Teaching Effectiveness

**DOI:** 10.7759/cureus.4290

**Published:** 2019-03-21

**Authors:** Matt Rutz, Joseph Turner, Katie Pettit, Megan M Palmer, Anthony Perkins, Dylan D Cooper

**Affiliations:** 1 Emergency Medicine, Indiana University School of Medicine, Indianapolis, USA; 2 Biostatistics, Indiana University School of Medicine, Indianapolis, USA

**Keywords:** residents as teachers, teaching effectiveness

## Abstract

Background

One of the key components of residency training is to become an educator. Resident physicians teach students, advanced practice providers, nurses, and even faculty on a daily basis.

Objective

The goal of this study was to identify the objective characteristics of residents, which correlate with perceived overall teaching effectiveness.

Methods

We conducted a one-year, retrospective study to identify factors that were associated with higher resident teaching evaluations. Senior emergency medicine (EM) teaching residents are evaluated by medical students following clinical teaching shifts. Eighteen factors pertaining to resident teaching effectiveness were chosen. Two items from the medical students' evaluations were analyzed against each factor: teaching effectiveness was measured on a five-point Likert scale and an overall teaching score (1-75).

Results

A total of 46 EM residents and 843 medical student evaluations were analyzed. The ACGME milestones for systems-based practice (*p* = 0.02) and accountability (*p* = 0.05) showed a statistically significant association with a rating of “five” on the Likert scale for teaching effectiveness. Three other ACGME milestones, systems-based practice (*p* = 0.01), task switching (*p* = 0.04), and team management (*p* = 0.03) also showed a statically significant association of receiving a score of 70 or greater on the overall teaching score.

Conclusion

Residents with higher performance associated with system management and accountability were perceived as highly effective teachers. USMLE and in-service exams were not predictive of higher teaching evaluations. Our data also suggest that effective teachers are working in both academic and community settings, providing a potential resource to academic departments and institutions.

## Introduction

Medical residents perform many roles during their training including learner, practitioner, and educator. As residents progress through training, the role of educator becomes more important. Teaching medical students, junior residents, and mid-level providers is often a daily occurrence for upper level residents [1]. While the importance of residents' role as teachers has at times been underemphasized, program directors are increasingly recognizing the importance of residents as teachers [2-3]. Further, medical students report that residents are often viewed as the most important educators [4].

A good clinical teacher has the ability to influence both a medical student’s performance and career choice. This has been demonstrated for both attending physicians and residents [5-6]. Exactly what makes a good clinical teacher needs further clarification. Sutkin and colleagues, in an extensive literature review on skills of effective clinical teachers, found the five core skills of clinical educators to be: medical knowledge, clinical skills, positive relationships with students in a supportive learning environment, communication skills, and enthusiasm [7]. When surveying medical students about the most important qualities in resident teachers, Melvin et al found approachability and respectfulness to be just below the strong knowledge base [8]. Through an extensive review of the literature and follow-up study, Ullian, Bland, and Simpson developed a model describing the four roles of clinical teachers [9]. These include:

1. The teacher role - being interested in teaching, making an effort to teach, being available and spending time with the resident, explaining, discussing and answering questions.

2. Instructor as a person - supportive, easy and fun to work with, helpful, and friendly.

3. The physician role - the clinical teacher is knowledgeable and clinically competent, is seen as a role model, has a good rapport with patients, and has an appropriate attitude.

4. The supervisor role - the clinical teacher gives the resident responsibility for patient care and opportunities to do the procedures, involves the learner, and reviews patients with learners.

This model has been used by others to study the effectiveness of clinical teachers [10].

Ultimately, as Sutkin states, “excellent teaching, although multifactorial, transcends ordinary teaching and is characterized by inspiring, supporting, actively involving, and communicating with students” [7]. Although many clinical teaching skills can be learned, there may be factors associated with the individual (e.g., demographic background, interest in academic medicine, strong communication skills) that result in some educators being more successful than others. In particular, it is unknown if objective measures exist that are associated with a resident’s ability to be a quality educator. As young physicians are evaluated constantly during residency through program-specific evaluations and Accreditation Council for Graduate Medical Education (ACGME) milestones, these measures may provide some predictive value as to who makes a great resident teacher in the eyes of medical students. The objective of this study was to determine what factors were associated with higher scores among medical student evaluations of resident educators.

## Materials and methods

We used a retrospective study design to identify residents' factors associated with evaluation scores as performed by medical students.

Participants included all residents in the Indiana University School of Medicine Emergency Medicine Residency Program (IUSMEM) who completed teaching shifts during the January 2014-January 2015 time period. At IUSMEM, third-year emergency medicine (EM) categorical residents, fourth-year emergency medicine-pediatrics (EM-Peds) residents, and fifth-year EM-Peds residents are scheduled for teaching shifts five days per week at one of two academic hospitals. Both hospitals are level 1 trauma centers with annual patient volumes of approximately 100,000 patients. They are staffed by the same faculty group and see similar patient populations. During these shifts, they typically supervise and educate 1-3 fourth-year medical students. Shifts are nine hours long and the resident’s primary responsibility is staffing medical student cases. Depending on the number of learners and patient volume, residents may also see their own primary patients.

When EM residents submit an evaluation for the students with whom they have worked in the E-value (Minneapolis, MN) system, an anonymous evaluation of the teaching resident is generated to be completed by the student. The evaluation questions were created by our institution and can be seen in Table 1. The specific questions examined for this study were selected because they map to at least one of the four roles of clinical teachers identified by Ullian, Bland, and Simpson [9], which is also included in Table 1.

**Table 1:** Medical student electronic evaluation mapped to four roles of the clinical teacher Roles as defined by Ullian, Bland, & Simpson [9]. Students were asked to respond to each of the following questions on a five-point Likert scale (1 = strongly disagree to 5 = strongly agree).

Role	Items on medical student evaluation of teaching residents
Teacher	Stated expectations clearly and concisely.
	Effectively elicited learners' knowledge of factual medical information.
	Expressed positive feedback.
	Gave corrective feedback.
	Offered learners suggestions for improvement.
Instructor as person	Expressed respect for learners.
	Listens carefully to connect with others.
	Inspires me to grow personally and professionally.
Physician	Patients and learners come to know him or her as both a good clinician and a caring person.
	Skillfully recognizes and supports emotions of patients, team, and self in difficult situations.
Supervisor	Encouraged learners to participate actively in discussion.
	Encouraged learners to bring up concerns.
	Effectively elicited learner's ability to apply medical knowledge to specific patients.
	Effectively elicited learners' ability to analyze or synthesize medical knowledge.

Each evaluation of a teaching resident by a student during the study period was extracted and entered into Microsoft Excel. 

Resident factors for comparison were chosen by study investigators and included gender; whether the resident was a chief resident; intended career (committed community practice, committed academics/fellowship, anticipated community practice, anticipated academics/fellowship); performance on United States Medical Licensing Exam (USMLE) step 1, USMLE step 2, first-year American Board of Emergency Medicine (ABEM) in-training, and second-year ABEM in-training exams; average level obtained during shift evaluations for seven of the ACGME Milestones (Table 2); both peer and nursing evaluations; and whether or not the resident was a participant in a voluntary “Academic Track” program designed by the residency program to help learners prepare for a career in academics. 

The authors chose the seven milestones listed in Table 2 because they evaluated the resident’s effectiveness in areas most important during the demands of a teaching shift. Residents are scored by faculty physicians on performance in the milestones following each shift as part of their normal evaluation process. Peer and nursing evaluations are an existing part of the resident’s evaluation during residency. They were not developed specifically for this study; however, the authors chose a question from each evaluation that was important during a teaching shift. Residents evaluated their peers (1-5 Likert scale) on the statement: “The resident is a strong communicator with patients, families, and other health care workers” with a “5” indicating strong agreement. They were evaluated by nursing (0-10 Likert scale) on the question “Does the resident effectively demonstrate team leadership skills?” with a “0” meaning never and a “10” meaning always.

**Table 2:** Selected Accreditation Council for Graduate Medical Education emergency medicine milestones PC, patient care; SBP, systems-based practice; PROF, professionalism; ICS, interpersonal and communication skills

Number	Milestone name
PC8	Task switching
SBP2	Systems-based management
SBP3	Technology
PROF1	Professional values
PROF2	Accountability
ICS1	Patient-centered communication
ICS2	Team management

Two outcomes of interest were extracted from the evaluation forms submitted by medical students for teaching residents. The first was a question asking about the resident's overall teaching effectiveness. The second was a total teaching score calculated from the sum of the values for overall teaching effectiveness and the 14 other questions on the teaching form (all items on 1-5 Likert scale). Each of the 18 resident factors listed in Tables 2-4 was compared to each of these two outcome measures for the association. For overall teaching effectiveness, we looked at an outcome of five versus any other score. For the total teaching score, we looked at dichotomous outcomes of scores less than or greater than/equal to 70. All testing was done using mixed effects models, including a random effect for the resident. 

All aspects of this study were reviewed and approved by the Institutional Review Board.

## Results

A total of 46 residents were evaluated during the January 2014-January 2015 time frame. There were 843 medical student evaluations for the 46 teaching residents. We analyzed 18 total variables for association with overall teaching effectiveness and total teaching score. Table 3 shows results for the dichotomous variables.

**Table 3:** Dichotomous variables with outcomes SD, standard deviation

	Mean effectiveness (SD)	P-value	Mean total score (SD)	P-value
Academic Track		0.743		0.790
No	4.62 (0.63)		69.68 (8.34)	
Yes	4.65 (0.56)		70.12 (7.43)	
Academic Job		0.865		0.504
Committed community	4.65 (0.64)		70.19 (8.23)	
Committed academic/fellowship	4.63 (0.58)		70.69 (6.31)	
Anticipated community	4.60 (0.65)		69.08 (8.66)	
Anticipated academic/fellowship	4.66 (0.58)		69.68 (8.17)	
Chief		0.255		0.256
No	4.60 (0.64)		69.44 (8.41)	
Yes	4.72 (0.52)		70.95 (6.82)	
Gender		0.921		0.724
Male	4.64 (0.61)		69.67 (8.03)	
Female	4.62 (0.63)		70.03 (8.15)	
Site		0.081		0.606
A	4.58 (0.68)		69.56 (8.81)	
B	4.68 (0,54)		70.06 (7.26)	

Table 4 shows results for the continuous variables. Two variables showed a statistically significant association with a teaching effectiveness of “five” on the Likert scale: milestone systems-based practice 2 (SBP2; *p* = 0.02), and milestone professionalism 1 (PROF1; *p* = 0.05). Three variables showed a statistically significant association with a total teaching score of 70 or greater: milestone patient care 8 (PC8; *p* = 0.04), systems-based practice 2 (SBP2; *p* = 0.01), and interpersonal and communications skills 2 (ICS2; *p* = 0.03).

**Table 4:** Correlations of continuous variables with outcomes USMLE, United States medical licensing exam; PC, patient care; SBP, systems-based practice; PROF, professionalism; ICS, interpersonal and communication skills; SD, standard deviation; *p* ≤ 0.05 statistically significant (*)

	Teaching effectiveness {Likert scale 1-5} (SD)		P-value	Total score {1-75} (SD)		P-value
	<5	5		<70	70+	
USMLE 1	235.2 (16.0)	233.6 (16.1)	0.386	235.7 (15.7)	233.3 (16.2)	0.145
USMLE 2	243.0 (14.2)	243.8 (15.0)	0.548	243.1 (14.6)	243.7 (14.9)	0.664
In-service 1	76.2 (5.3)	76.4 (5.3)	0.517	76.3 (5.4)	76.4 (5.2)	0.678
In-service 2	81.1 (5.9)	80.6 (6.3)	0.415	81.0 (5.9)	80.6 (6.3)	0.574
PC8	3.88 (0.3)	3.93 (0.3)	0.059	3.87 (0.3)	3.93 (0.3)	0.043*
SBP2	3.76 (0.2)	3.81 (0.2)	0.020*	3.76 (0.2)	3.82 (0.2)	0.013*
SBP3	3.81 (0.2)	3.83 (0.2)	0.157	3.81 (0.2)	3.83 (0.2)	0.233
PROF1	3.79 (0.2)	3.82 (0.2)	0.052*	3.80 (0.2)	3.82 (0.2)	0.312
PROF2	3.70 (0.2)	3.69 (0.3)	0.841	3.63 (0.3)	3.70 (0.3)	0.447
ICS1	3.81 (0.3)	3.83 (0.3)	0.458	3.80 (0.3)	3.83 (0.3)	0.089
ICS2	3.72 (0.2)	3.75 (0.3)	0.268	3.71 (0.2)	3.76 (0.3)	0.030*
Peer	4.69 (0.2)	4.71 (0.2)	0.166	4.69 (0.2)	4.71 (0.2)	0.269
Nursing (1-10)	8.88 (1.0)	8.86 (1.1)	0.522	8.87 (1.0)	8.83 (1.1)	0.758

There was no association of a teaching resident’s overall effectiveness or total teaching score with their USMLE step one or two scores, in-service scores, peer evaluations, nursing evaluations, or their performance on the other EM Milestones that were analyzed. 

Whether the teaching resident was male or female, was a chief resident, or participated in the residency’s academic track was also not associated with increased teaching effectiveness as evaluated by the medical student. The residents split their teaching shifts between two academic teaching hospitals, but the site was not associated with a difference in teaching effectiveness.

Finally, the residents that are now practicing at or are intending to start in an academic setting did not receive better teaching evaluations than those residents joining a community practice.

## Discussion

Attending physicians and residents teach medical students, advanced practice providers, nurses, and even other physicians on a daily basis. The positive impact of attendings and resident as teachers on medical students exam scores and clinical performance has been studied previously [5-6,11]. Cooper et al. showed that during simulation debriefings, residents were perceived to be as effective as emergency medicine faculty with regard to education [12]. This study was an attempt to identify objective measures associated with effective resident teachers.

Our findings suggest that medical students perceived those residents who can manage flow in the emergency department (SBP2) and demonstrate compassion, integrity, and respect for others (PROF1) as having the greatest teaching effectiveness. While the reason for the association between SBP2 and teaching effectiveness is not clear, there is well-documented tension between education and service missions in medical education [13]. One possibility is that residents who can manage flow in two busy academic emergency departments have organizational and time management skills that allowed them to balance these missions and be available to guide medical students in patient management while providing quality teaching. Our findings support surveys done by Melvin et al. regarding medical students’ appreciation for respectfulness [8]. It is not surprising that medical students who perceived residents as more respectful were also judged to be better teachers. These findings were reinforced in the overall teaching ratings (≥ 70/75) when residents had higher ACGME milestone ratings in task switching (PC8), team management (ICS2), and departmental flow (SBP2). 

Interestingly, residents who identified themselves as going into community practice were viewed as equally effective teachers as those going into academic practice. This has a couple of important implications for medical education. First, it suggests institutions could engage the potentially large pool of effective teachers in community practice as adjunct faculty for student and resident education. Secondly, it suggests that community physicians have the skills to effectively educate members of other healthcare professions with whom they frequently interact.

Test performance both in the form the USMLE and NBME did not correspond to a significantly higher rating in overall teaching or teaching effectiveness. This is in contrast to other studies that have found that a strong knowledge base was highly rated among medical students when surveyed [8]. There are several hypotheses for this result. During an emergency medicine clerkship, medical students may not perceive the varying levels of the knowledge base in their resident teachers. Medical students may feel more uncomfortable by the multitasking and multiple patient management skills and prefer residents that can help them manage this stressor as opposed to lack of knowledge. This also may be a reflection more of the confidence (or overconfidence) in which a teacher speaks with students. Medical students may confuse confidence with knowledge

Our study has several limitations. The study was done at a single academic center; however, our fourth-year emergency medicine clerkship is one of the largest in the country. This may improve generalizability that would otherwise have been lost in a smaller academic center. Student ratings may not be a comprehensive measure of actual teaching effectiveness, but rather, reflect perceived teaching effectiveness. The overall high ratings of the teaching residents may limit the ability to detect any differences. Finally, the study focused on emergency medicine residents and may not be generalizable to residents in other specialties. 

## Conclusions

Our study found that residents with higher performance on milestones associated with system management and accountability were overall perceived as highly effective teachers. USMLE and in-service exams were not predictive of higher teaching evaluations, suggesting that those with the best medical knowledge may not be the best teachers. Finally, residents intending to pursue community practice were perceived as equally effective teachers to those staying in academics. Future studies should focus on more objective markers of teaching effectiveness. 
